# 3,5-Bis(4-meth­oxy­phen­yl)-4*H*-1,2,4-triazol-4-amine

**DOI:** 10.1107/S1600536810030898

**Published:** 2010-08-11

**Authors:** Hui Yang, Zhan-Dong Huang, Guang Yang, Seik Weng Ng

**Affiliations:** a Department of Chemistry, Zhengzhou University, Zhengzhou 450001, People’s Republic of China; b Department of Chemistry, University of Malaya, 50603 Kuala Lumpur, Malaysia

## Abstract

The title compound, C_16_H_16_N_4_O_2_, crystallizes with two mol­ecules in the asymmetric unit, which are related by a non-crystallographic centre of inversion. The phenyl­ene rings are twisted out of the mean plane of the triazole ring by 19.3 (1) and 21.4 (1)° for one independent mol­ecule and by 16.3 (1) and 18.1 (1)° for the other mol­ecule. In the crystal, adjacent mol­ecules are linked by amine–triazole N—H⋯N hydrogen bonds, forming chains running along the *a* axis.

## Related literature

For the synthesis, see: Bentiss *et al.* (1999 ▶). For the two polymorphs of 3,5-diphenyl-1,2,4-triazol-4-amine, see: Ikemi *et al.* (2002 ▶); Zhang *et al.* (2009 ▶).
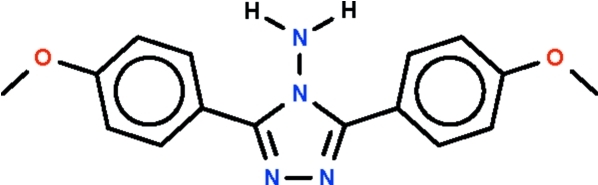


## Experimental

### 

#### Crystal data

C_16_H_16_N_4_O_2_
*M* *_r_* = 296.33
Monoclinic, 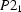

*a* = 11.2232 (9) Å
*b* = 7.2386 (6) Å
*c* = 17.9766 (14) Å
β = 107.147 (1)°
*V* = 1395.51 (19) Å^3^
*Z* = 4
Mo *K*α radiation
μ = 0.10 mm^−1^
*T* = 100 K
0.40 × 0.20 × 0.10 mm

#### Data collection

Bruker SMART APEX diffractometer
12717 measured reflections
3459 independent reflections
2709 reflections with *I* > 2σ(*I*)
*R*_int_ = 0.086

#### Refinement

*R*[*F* ^2^ > 2σ(*F* ^2^)] = 0.054
*wR*(*F* ^2^) = 0.147
*S* = 1.06
3459 reflections
417 parameters
5 restraints
H atoms treated by a mixture of independent and constrained refinement
Δρ_max_ = 0.48 e Å^−3^
Δρ_min_ = −0.29 e Å^−3^

### 

Data collection: *APEX2* (Bruker, 2009 ▶); cell refinement: *SAINT* (Bruker, 2009 ▶); data reduction: *SAINT*; program(s) used to solve structure: *SHELXS97* (Sheldrick, 2008 ▶); program(s) used to refine structure: *SHELXL97* (Sheldrick, 2008 ▶); molecular graphics: *X-SEED* (Barbour, 2001 ▶); software used to prepare material for publication: *publCIF* (Westrip, 2010 ▶).

## Supplementary Material

Crystal structure: contains datablocks global, I. DOI: 10.1107/S1600536810030898/bt5318sup1.cif

Structure factors: contains datablocks I. DOI: 10.1107/S1600536810030898/bt5318Isup2.hkl

Additional supplementary materials:  crystallographic information; 3D view; checkCIF report

## Figures and Tables

**Table 1:** Hydrogen-bond geometry (Å, °)

*D*—H⋯*A*	*D*—H	H⋯*A*	*D*⋯*A*	*D*—H⋯*A*
N1—H1⋯N8	0.86 (4)	2.20 (2)	3.027 (5)	162 (4)
N5—H3⋯N4^i^	0.86 (3)	2.18 (3)	3.029 (5)	166 (3)

Symmetry code: (i) 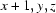.

**Fig. 1.**
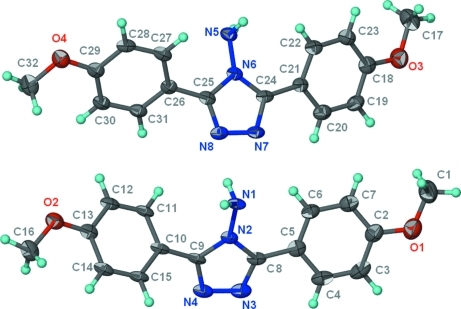
Anisotropic displacement ellipsoid plot (Barbour, 2001) of the two independent molecules of C16H16N4O2 at the 70% probability level; hydrogen atoms are drawn as spheres of arbitrary radius.

**Fig. 2.**
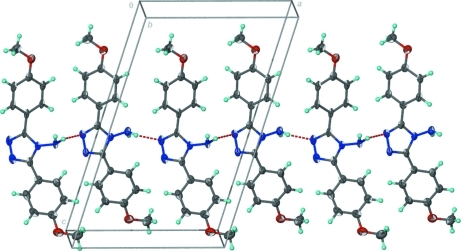
Hydrogen-bonded chain motif.

## supplementary crystallographic information

### Comment

The compound was intended for the synthesis of gold complexes as the two nitrogen atoms of the ring can possibly give rise to triangular-shaped trinuclear compounds. The parent compound, 3,5-diphenyl-1,2,4-triazol-4-amine, which exists in two polymorphic forms (Ikemi *et al.*, 2002; Zhang *et al.*, 2009), furnishes a number of metal derivatives. In the methoxy-substituted derivative (Scheme I), the five-membered triazole ring is planar but the phenylene rings are twisted out of the mean plane [19.3 (1), 21.4 (1) °; 16.3 (1), 18.1 (1) °] in the two indepedent molecules (Fig. 1). The twist angles are smaller than those noted in the parent compound. Adjacent molecules are linked by *N*–H_amino_···N_triazole_ hydrogen bonds to form chain running along the *a*-axis (Fig. 2).

### Experimental

The compound was synthesized by using a literature method (Bentiss *et al.*, 1999), and crystals were obtained upon recrystallzation from aqueous methanol.

### Refinement

Carbon-bound H-atoms were placed in calculated positions (C—H 0.95 to 0.98 Å) and were included in the refinement in the riding model approximation with *U*(H) set to 1.2 to 1.5*U*(C).

The amino H-atoms were located in a difference Fourier map, and were refined with a distance restraint of N–H 0.86±0.01 Å; their displacement parameters were freely refined. The displacement parameters of the hydrogen atoms involved in hydrogen bonding are somewhat smaller than those not involved in hydrogen bonding.

2352 Friedel pairs were merged.

### Crystal data

**Table:**

C_16_H_16_N_4_O_2_	*F*(000) = 624
*M**_r_* = 296.33	*D*_x_ = 1.410 Mg m^−^^3^
Monoclinic, *P*2_1_	Mo *K*α radiation, λ = 0.71073 Å
Hall symbol: P 2yb	Cell parameters from 5135 reflections
*a* = 11.2232 (9) Å	θ = 2.4–28.0°
*b* = 7.2386 (6) Å	µ = 0.10 mm^−^^1^
*c* = 17.9766 (14) Å	*T* = 100 K
β = 107.147 (1)°	Block, colorless
*V* = 1395.51 (19) Å^3^	0.40 × 0.20 × 0.10 mm
*Z* = 4	

### Data collection

**Table:**

Bruker SMART APEX diffractometer	2709 reflections with *I* > 2σ(*I*)
Radiation source: fine-focus sealed tube	*R*_int_ = 0.086
graphite	θ_max_ = 27.5°, θ_min_ = 1.9°
ω scans	*h* = −13→14
12717 measured reflections	*k* = −8→9
3459 independent reflections	*l* = −23→23

### Refinement

**Table:**

Refinement on *F*^2^	Primary atom site location: structure-invariant direct methods
Least-squares matrix: full	Secondary atom site location: difference Fourier map
*R*[*F*^2^ > 2σ(*F*^2^)] = 0.054	Hydrogen site location: inferred from neighbouring sites
*wR*(*F*^2^) = 0.147	H atoms treated by a mixture of independent and constrained refinement
*S* = 1.06	*w* = 1/[σ^2^(*F*_o_^2^) + (0.0797*P*)^2^ + 0.357*P*] where *P* = (*F*_o_^2^ + 2*F*_c_^2^)/3
3459 reflections	(Δ/σ)_max_ = 0.001
417 parameters	Δρ_max_ = 0.48 e Å^−^^3^
5 restraints	Δρ_min_ = −0.29 e Å^−^^3^

### Fractional atomic coordinates and isotropic or equivalent isotropic displacement parameters (Å^2^)

**Table:**

	*x*	*y*	*z*	*U*_iso_*/*U*_eq_	
O1	0.9153 (3)	0.5009 (5)	0.97018 (15)	0.0398 (8)	
O2	0.3820 (2)	0.3666 (4)	0.22405 (14)	0.0290 (6)	
O3	1.3936 (3)	−0.0328 (5)	0.95364 (14)	0.0361 (7)	
O4	0.8588 (3)	0.1022 (4)	0.20680 (14)	0.0305 (6)	
N1	0.7126 (3)	0.4643 (4)	0.58797 (17)	0.0204 (6)	
H1	0.760 (3)	0.391 (5)	0.572 (2)	0.035 (12)*	
H2	0.690 (4)	0.541 (5)	0.5496 (19)	0.047 (14)*	
N2	0.6066 (3)	0.3855 (4)	0.60149 (16)	0.0195 (6)	
N3	0.4738 (3)	0.3072 (5)	0.66424 (18)	0.0271 (7)	
N4	0.4144 (3)	0.2941 (5)	0.58558 (18)	0.0250 (7)	
N5	1.1929 (3)	0.0393 (5)	0.56489 (17)	0.0236 (7)	
H3	1.250 (3)	0.124 (4)	0.5753 (19)	0.016 (9)*	
H4	1.218 (4)	−0.062 (4)	0.590 (2)	0.048 (15)*	
N6	1.0885 (3)	0.1032 (4)	0.58499 (16)	0.0188 (6)	
N7	0.9526 (3)	0.1627 (4)	0.64705 (17)	0.0222 (7)	
N8	0.8932 (3)	0.1714 (4)	0.56805 (16)	0.0214 (6)	
C1	1.0453 (4)	0.5275 (8)	0.9815 (2)	0.0457 (12)	
H1A	1.0859	0.5625	1.0358	0.069*	
H1B	1.0573	0.6257	0.9469	0.069*	
H1C	1.0821	0.4124	0.9697	0.069*	
C2	0.8434 (4)	0.4603 (6)	0.8967 (2)	0.0297 (9)	
C3	0.7162 (4)	0.4492 (6)	0.8863 (2)	0.0329 (9)	
H3A	0.6851	0.4625	0.9298	0.039*	
C4	0.6349 (4)	0.4191 (6)	0.8137 (2)	0.0300 (9)	
H4A	0.5478	0.4138	0.8069	0.036*	
C5	0.6804 (4)	0.3960 (5)	0.7490 (2)	0.0244 (8)	
C6	0.8074 (4)	0.3992 (5)	0.7609 (2)	0.0253 (8)	
H6	0.8392	0.3781	0.7182	0.030*	
C7	0.8894 (4)	0.4322 (5)	0.8337 (2)	0.0281 (9)	
H7	0.9767	0.4358	0.8406	0.034*	
C8	0.5891 (3)	0.3635 (5)	0.6732 (2)	0.0215 (8)	
C9	0.4956 (3)	0.3413 (5)	0.5486 (2)	0.0199 (7)	
C10	0.4680 (3)	0.3512 (5)	0.4645 (2)	0.0203 (8)	
C11	0.5510 (3)	0.2879 (5)	0.4247 (2)	0.0206 (7)	
H11	0.6301	0.2400	0.4533	0.025*	
C12	0.5189 (3)	0.2948 (5)	0.3459 (2)	0.0234 (8)	
H12	0.5756	0.2500	0.3200	0.028*	
C13	0.4049 (4)	0.3657 (5)	0.3023 (2)	0.0232 (8)	
C14	0.3203 (3)	0.4288 (5)	0.3405 (2)	0.0235 (8)	
H14	0.2412	0.4757	0.3115	0.028*	
C15	0.3530 (3)	0.4220 (5)	0.4202 (2)	0.0219 (8)	
H15	0.2961	0.4666	0.4461	0.026*	
C16	0.2587 (4)	0.4115 (6)	0.1774 (2)	0.0342 (9)	
H16A	0.2526	0.3960	0.1223	0.051*	
H16B	0.2400	0.5399	0.1871	0.051*	
H16C	0.1988	0.3293	0.1910	0.051*	
C17	1.5222 (4)	−0.0665 (7)	0.9644 (2)	0.0410 (11)	
H17A	1.5601	−0.1173	1.0166	0.062*	
H17B	1.5637	0.0496	0.9589	0.062*	
H17C	1.5314	−0.1551	0.9253	0.062*	
C18	1.3227 (4)	0.0143 (5)	0.8802 (2)	0.0266 (8)	
C19	1.1953 (4)	0.0294 (6)	0.8700 (2)	0.0272 (8)	
H19	1.1632	0.0146	0.9130	0.033*	
C20	1.1158 (4)	0.0662 (6)	0.7968 (2)	0.0266 (9)	
H20	1.0287	0.0750	0.7900	0.032*	
C21	1.1598 (3)	0.0907 (5)	0.73283 (19)	0.0204 (7)	
C22	1.2876 (3)	0.0831 (5)	0.7455 (2)	0.0224 (8)	
H22	1.3202	0.1051	0.7032	0.027*	
C23	1.3697 (3)	0.0442 (6)	0.8186 (2)	0.0256 (8)	
H23	1.4570	0.0383	0.8258	0.031*	
C24	1.0701 (3)	0.1204 (5)	0.65715 (19)	0.0196 (7)	
C25	0.9752 (3)	0.1338 (5)	0.53199 (19)	0.0203 (7)	
C26	0.9471 (3)	0.1218 (5)	0.44711 (18)	0.0206 (7)	
C27	1.0301 (3)	0.1839 (5)	0.4078 (2)	0.0227 (8)	
H27	1.1091	0.2324	0.4362	0.027*	
C28	0.9970 (3)	0.1744 (5)	0.3280 (2)	0.0224 (8)	
H28	1.0538	0.2149	0.3013	0.027*	
C29	0.8810 (3)	0.1060 (5)	0.2859 (2)	0.0229 (8)	
C30	0.7986 (3)	0.0431 (5)	0.3239 (2)	0.0235 (8)	
H30	0.7197	−0.0053	0.2953	0.028*	
C31	0.8327 (3)	0.0519 (5)	0.4042 (2)	0.0223 (8)	
H31	0.7762	0.0089	0.4306	0.027*	
C32	0.7358 (4)	0.0492 (6)	0.1612 (2)	0.0339 (9)	
H32A	0.7269	0.0688	0.1059	0.051*	
H32B	0.6740	0.1241	0.1763	0.051*	
H32C	0.7223	−0.0817	0.1702	0.051*	

### Atomic displacement parameters (Å^2^)

**Table:**

	*U*^11^	*U*^22^	*U*^33^	*U*^12^	*U*^13^	*U*^23^
O1	0.0458 (19)	0.0434 (19)	0.0300 (13)	0.0051 (16)	0.0107 (12)	−0.0016 (13)
O2	0.0240 (15)	0.0265 (15)	0.0349 (14)	0.0012 (12)	0.0061 (11)	−0.0008 (11)
O3	0.0378 (17)	0.0413 (18)	0.0283 (13)	−0.0032 (15)	0.0083 (12)	0.0034 (12)
O4	0.0288 (15)	0.0290 (15)	0.0321 (13)	−0.0016 (13)	0.0067 (11)	−0.0003 (12)
N1	0.0146 (14)	0.0163 (15)	0.0330 (15)	−0.0016 (13)	0.0115 (12)	0.0035 (12)
N2	0.0169 (15)	0.0122 (14)	0.0310 (14)	0.0003 (12)	0.0095 (12)	−0.0001 (11)
N3	0.0259 (17)	0.0195 (17)	0.0400 (17)	0.0034 (14)	0.0161 (14)	0.0045 (13)
N4	0.0193 (15)	0.0161 (15)	0.0420 (17)	0.0007 (13)	0.0129 (13)	0.0016 (12)
N5	0.0175 (16)	0.0187 (15)	0.0342 (16)	0.0003 (13)	0.0071 (13)	−0.0053 (13)
N6	0.0170 (14)	0.0119 (13)	0.0279 (14)	−0.0010 (12)	0.0072 (12)	−0.0005 (11)
N7	0.0174 (15)	0.0168 (15)	0.0349 (15)	0.0008 (13)	0.0117 (13)	−0.0029 (12)
N8	0.0212 (15)	0.0140 (14)	0.0320 (15)	0.0008 (12)	0.0128 (12)	−0.0001 (12)
C1	0.043 (3)	0.049 (3)	0.037 (2)	0.008 (2)	−0.0003 (19)	−0.003 (2)
C2	0.037 (2)	0.025 (2)	0.0283 (18)	0.0084 (19)	0.0109 (16)	0.0026 (15)
C3	0.043 (3)	0.030 (2)	0.0323 (19)	0.009 (2)	0.0218 (18)	0.0051 (16)
C4	0.030 (2)	0.025 (2)	0.041 (2)	0.0064 (17)	0.0205 (17)	0.0065 (16)
C5	0.030 (2)	0.0135 (17)	0.0315 (18)	0.0014 (16)	0.0116 (15)	0.0022 (14)
C6	0.027 (2)	0.0213 (19)	0.0315 (18)	0.0030 (17)	0.0139 (15)	−0.0002 (15)
C7	0.030 (2)	0.024 (2)	0.0325 (18)	0.0040 (17)	0.0126 (16)	0.0032 (15)
C8	0.0208 (18)	0.0112 (17)	0.0364 (19)	0.0009 (15)	0.0143 (15)	0.0032 (14)
C9	0.0110 (17)	0.0084 (16)	0.0411 (19)	0.0004 (13)	0.0087 (15)	−0.0014 (13)
C10	0.0124 (17)	0.0064 (16)	0.043 (2)	−0.0001 (13)	0.0100 (15)	−0.0018 (13)
C11	0.0109 (16)	0.0119 (17)	0.0392 (18)	−0.0009 (13)	0.0074 (14)	−0.0003 (14)
C12	0.0146 (17)	0.0164 (18)	0.0401 (19)	−0.0001 (14)	0.0093 (15)	−0.0012 (14)
C13	0.0207 (18)	0.0111 (16)	0.0369 (19)	−0.0018 (15)	0.0072 (15)	−0.0025 (14)
C14	0.0141 (17)	0.0119 (18)	0.043 (2)	0.0008 (14)	0.0071 (15)	0.0015 (14)
C15	0.0131 (17)	0.0086 (17)	0.046 (2)	0.0016 (14)	0.0112 (15)	−0.0031 (14)
C16	0.033 (2)	0.025 (2)	0.039 (2)	0.0030 (18)	0.0005 (17)	−0.0027 (16)
C17	0.033 (2)	0.044 (3)	0.040 (2)	−0.003 (2)	0.0010 (18)	0.0060 (19)
C18	0.030 (2)	0.0184 (19)	0.0311 (18)	−0.0020 (16)	0.0095 (15)	−0.0015 (14)
C19	0.031 (2)	0.028 (2)	0.0274 (17)	−0.0030 (18)	0.0165 (16)	−0.0002 (15)
C20	0.028 (2)	0.020 (2)	0.0357 (19)	−0.0008 (16)	0.0143 (16)	−0.0042 (14)
C21	0.0208 (18)	0.0141 (17)	0.0275 (16)	−0.0027 (15)	0.0089 (14)	−0.0025 (13)
C22	0.0226 (18)	0.0151 (18)	0.0323 (17)	−0.0042 (15)	0.0122 (15)	−0.0004 (13)
C23	0.0231 (19)	0.0211 (19)	0.0328 (18)	−0.0002 (16)	0.0086 (15)	−0.0013 (15)
C24	0.0184 (17)	0.0100 (16)	0.0336 (17)	−0.0021 (14)	0.0129 (14)	−0.0017 (13)
C25	0.0173 (17)	0.0122 (18)	0.0310 (17)	−0.0014 (14)	0.0067 (14)	0.0005 (13)
C26	0.0210 (18)	0.0126 (17)	0.0278 (16)	0.0065 (15)	0.0065 (14)	0.0007 (13)
C27	0.0171 (18)	0.0117 (17)	0.0383 (19)	0.0009 (14)	0.0066 (15)	−0.0001 (14)
C28	0.0206 (18)	0.0136 (17)	0.0365 (19)	0.0004 (15)	0.0140 (15)	0.0012 (14)
C29	0.0217 (18)	0.0137 (17)	0.0326 (17)	0.0046 (15)	0.0067 (15)	−0.0008 (14)
C30	0.0186 (18)	0.0157 (18)	0.0356 (18)	0.0011 (15)	0.0070 (14)	−0.0034 (14)
C31	0.0171 (17)	0.0157 (18)	0.0346 (18)	0.0027 (15)	0.0084 (15)	−0.0009 (14)
C32	0.035 (2)	0.028 (2)	0.0340 (19)	−0.0001 (19)	0.0028 (16)	0.0022 (16)

### Geometric parameters (Å, °)

**Table:**

O1—C2	1.361 (4)	C10—C11	1.408 (5)
O1—C1	1.426 (5)	C11—C12	1.357 (5)
O2—C13	1.353 (4)	C11—H11	0.9500
O2—C16	1.428 (5)	C12—C13	1.386 (5)
O3—C18	1.368 (4)	C12—H12	0.9500
O3—C17	1.419 (5)	C13—C14	1.403 (5)
O4—C29	1.370 (4)	C14—C15	1.372 (5)
O4—C32	1.435 (5)	C14—H14	0.9500
N1—N2	1.404 (4)	C15—H15	0.9500
N1—H1	0.86 (4)	C16—H16A	0.9800
N1—H2	0.86 (3)	C16—H16B	0.9800
N2—C9	1.363 (4)	C16—H16C	0.9800
N2—C8	1.368 (4)	C17—H17A	0.9800
N3—C8	1.321 (5)	C17—H17B	0.9800
N3—N4	1.377 (4)	C17—H17C	0.9800
N4—C9	1.321 (5)	C18—C23	1.377 (5)
N5—N6	1.403 (4)	C18—C19	1.392 (5)
N5—H3	0.86 (3)	C19—C20	1.380 (5)
N5—H4	0.87 (3)	C19—H19	0.9500
N6—C25	1.363 (4)	C20—C21	1.391 (5)
N6—C24	1.378 (4)	C20—H20	0.9500
N7—C24	1.314 (5)	C21—C22	1.386 (5)
N7—N8	1.381 (4)	C21—C24	1.450 (5)
N8—C25	1.301 (5)	C22—C23	1.393 (5)
C1—H1A	0.9800	C22—H22	0.9500
C1—H1B	0.9800	C23—H23	0.9500
C1—H1C	0.9800	C25—C26	1.467 (4)
C2—C3	1.387 (6)	C26—C31	1.384 (5)
C2—C7	1.392 (5)	C26—C27	1.398 (5)
C3—C4	1.372 (6)	C27—C28	1.374 (5)
C3—H3A	0.9500	C27—H27	0.9500
C4—C5	1.411 (5)	C28—C29	1.390 (5)
C4—H4A	0.9500	C28—H28	0.9500
C5—C6	1.378 (5)	C29—C30	1.379 (5)
C5—C8	1.463 (5)	C30—C31	1.382 (5)
C6—C7	1.380 (5)	C30—H30	0.9500
C6—H6	0.9500	C31—H31	0.9500
C7—H7	0.9500	C32—H32A	0.9800
C9—C10	1.454 (5)	C32—H32B	0.9800
C10—C15	1.398 (5)	C32—H32C	0.9800
			
C2—O1—C1	117.1 (3)	C13—C14—H14	120.4
C13—O2—C16	117.7 (3)	C14—C15—C10	121.7 (3)
C18—O3—C17	117.0 (3)	C14—C15—H15	119.2
C29—O4—C32	116.6 (3)	C10—C15—H15	119.2
N2—N1—H1	116 (3)	O2—C16—H16A	109.5
N2—N1—H2	109 (3)	O2—C16—H16B	109.5
H1—N1—H2	101 (4)	H16A—C16—H16B	109.5
C9—N2—C8	105.9 (3)	O2—C16—H16C	109.5
C9—N2—N1	128.4 (3)	H16A—C16—H16C	109.5
C8—N2—N1	125.0 (3)	H16B—C16—H16C	109.5
C8—N3—N4	107.8 (3)	O3—C17—H17A	109.5
C9—N4—N3	107.6 (3)	O3—C17—H17B	109.5
N6—N5—H3	109 (3)	H17A—C17—H17B	109.5
N6—N5—H4	108 (3)	O3—C17—H17C	109.5
H3—N5—H4	113 (4)	H17A—C17—H17C	109.5
C25—N6—C24	106.0 (3)	H17B—C17—H17C	109.5
C25—N6—N5	123.5 (3)	O3—C18—C23	124.3 (4)
C24—N6—N5	129.8 (3)	O3—C18—C19	115.5 (3)
C24—N7—N8	108.2 (3)	C23—C18—C19	120.2 (3)
C25—N8—N7	107.8 (3)	C20—C19—C18	119.5 (3)
O1—C1—H1A	109.5	C20—C19—H19	120.3
O1—C1—H1B	109.5	C18—C19—H19	120.3
H1A—C1—H1B	109.5	C19—C20—C21	121.7 (4)
O1—C1—H1C	109.5	C19—C20—H20	119.2
H1A—C1—H1C	109.5	C21—C20—H20	119.2
H1B—C1—H1C	109.5	C22—C21—C20	117.5 (3)
O1—C2—C3	115.8 (3)	C22—C21—C24	124.0 (3)
O1—C2—C7	124.5 (4)	C20—C21—C24	118.5 (3)
C3—C2—C7	119.8 (3)	C21—C22—C23	121.9 (3)
C4—C3—C2	120.4 (3)	C21—C22—H22	119.0
C4—C3—H3A	119.8	C23—C22—H22	119.0
C2—C3—H3A	119.8	C18—C23—C22	119.1 (4)
C3—C4—C5	120.2 (4)	C18—C23—H23	120.5
C3—C4—H4A	119.9	C22—C23—H23	120.5
C5—C4—H4A	119.9	N7—C24—N6	108.3 (3)
C6—C5—C4	118.6 (3)	N7—C24—C21	123.7 (3)
C6—C5—C8	123.8 (3)	N6—C24—C21	128.0 (3)
C4—C5—C8	117.6 (4)	N8—C25—N6	109.7 (3)
C5—C6—C7	121.4 (3)	N8—C25—C26	124.4 (3)
C5—C6—H6	119.3	N6—C25—C26	126.0 (3)
C7—C6—H6	119.3	C31—C26—C27	118.8 (3)
C6—C7—C2	119.5 (4)	C31—C26—C25	118.7 (3)
C6—C7—H7	120.2	C27—C26—C25	122.4 (3)
C2—C7—H7	120.2	C28—C27—C26	119.8 (3)
N3—C8—N2	109.2 (3)	C28—C27—H27	120.1
N3—C8—C5	123.7 (3)	C26—C27—H27	120.1
N2—C8—C5	127.1 (3)	C27—C28—C29	120.5 (4)
N4—C9—N2	109.5 (3)	C27—C28—H28	119.7
N4—C9—C10	124.7 (3)	C29—C28—H28	119.7
N2—C9—C10	125.8 (3)	O4—C29—C30	124.4 (3)
C15—C10—C11	118.0 (3)	O4—C29—C28	115.3 (3)
C15—C10—C9	119.2 (3)	C30—C29—C28	120.3 (3)
C11—C10—C9	122.8 (3)	C29—C30—C31	118.9 (3)
C12—C11—C10	120.4 (3)	C29—C30—H30	120.6
C12—C11—H11	119.8	C31—C30—H30	120.6
C10—C11—H11	119.8	C30—C31—C26	121.7 (4)
C11—C12—C13	121.4 (4)	C30—C31—H31	119.2
C11—C12—H12	119.3	C26—C31—H31	119.2
C13—C12—H12	119.3	O4—C32—H32A	109.5
O2—C13—C12	116.8 (3)	O4—C32—H32B	109.5
O2—C13—C14	124.0 (3)	H32A—C32—H32B	109.5
C12—C13—C14	119.3 (3)	O4—C32—H32C	109.5
C15—C14—C13	119.2 (3)	H32A—C32—H32C	109.5
C15—C14—H14	120.4	H32B—C32—H32C	109.5
			
C8—N3—N4—C9	0.5 (4)	C9—C10—C15—C14	−177.7 (3)
C24—N7—N8—C25	0.2 (4)	C17—O3—C18—C23	−5.2 (6)
C1—O1—C2—C3	−175.3 (4)	C17—O3—C18—C19	174.2 (4)
C1—O1—C2—C7	4.1 (6)	O3—C18—C19—C20	−176.4 (4)
O1—C2—C3—C4	176.4 (4)	C23—C18—C19—C20	3.0 (6)
C7—C2—C3—C4	−3.0 (7)	C18—C19—C20—C21	−0.7 (6)
C2—C3—C4—C5	1.1 (7)	C19—C20—C21—C22	−2.1 (6)
C3—C4—C5—C6	1.8 (6)	C19—C20—C21—C24	177.2 (4)
C3—C4—C5—C8	179.7 (4)	C20—C21—C22—C23	2.8 (5)
C4—C5—C6—C7	−2.8 (6)	C24—C21—C22—C23	−176.4 (4)
C8—C5—C6—C7	179.4 (4)	O3—C18—C23—C22	177.1 (4)
C5—C6—C7—C2	1.0 (6)	C19—C18—C23—C22	−2.3 (6)
O1—C2—C7—C6	−177.4 (4)	C21—C22—C23—C18	−0.7 (6)
C3—C2—C7—C6	2.0 (6)	N8—N7—C24—N6	0.6 (4)
N4—N3—C8—N2	−0.6 (4)	N8—N7—C24—C21	−177.0 (3)
N4—N3—C8—C5	179.1 (3)	C25—N6—C24—N7	−1.2 (4)
C9—N2—C8—N3	0.5 (4)	N5—N6—C24—N7	−171.7 (4)
N1—N2—C8—N3	172.0 (3)	C25—N6—C24—C21	176.3 (4)
C9—N2—C8—C5	−179.3 (4)	N5—N6—C24—C21	5.8 (6)
N1—N2—C8—C5	−7.7 (6)	C22—C21—C24—N7	−166.4 (4)
C6—C5—C8—N3	159.5 (4)	C20—C21—C24—N7	14.3 (6)
C4—C5—C8—N3	−18.4 (5)	C22—C21—C24—N6	16.5 (6)
C6—C5—C8—N2	−20.8 (6)	C20—C21—C24—N6	−162.8 (4)
C4—C5—C8—N2	161.4 (4)	N7—N8—C25—N6	−1.0 (4)
N3—N4—C9—N2	−0.2 (4)	N7—N8—C25—C26	177.4 (3)
N3—N4—C9—C10	−177.9 (3)	C24—N6—C25—N8	1.4 (4)
C8—N2—C9—N4	−0.2 (4)	N5—N6—C25—N8	172.6 (3)
N1—N2—C9—N4	−171.3 (3)	C24—N6—C25—C26	−177.0 (3)
C8—N2—C9—C10	177.4 (3)	N5—N6—C25—C26	−5.8 (6)
N1—N2—C9—C10	6.3 (6)	N8—C25—C26—C31	−35.0 (5)
N4—C9—C10—C15	39.4 (5)	N6—C25—C26—C31	143.2 (4)
N2—C9—C10—C15	−137.9 (4)	N8—C25—C26—C27	142.9 (4)
N4—C9—C10—C11	−138.9 (4)	N6—C25—C26—C27	−38.9 (6)
N2—C9—C10—C11	43.8 (5)	C31—C26—C27—C28	0.0 (5)
C15—C10—C11—C12	−0.5 (5)	C25—C26—C27—C28	−177.9 (3)
C9—C10—C11—C12	177.8 (3)	C26—C27—C28—C29	0.9 (5)
C10—C11—C12—C13	0.8 (5)	C32—O4—C29—C30	7.6 (5)
C16—O2—C13—C12	170.4 (3)	C32—O4—C29—C28	−174.2 (3)
C16—O2—C13—C14	−8.6 (5)	C27—C28—C29—O4	−179.6 (3)
C11—C12—C13—O2	179.9 (3)	C27—C28—C29—C30	−1.3 (6)
C11—C12—C13—C14	−1.1 (5)	O4—C29—C30—C31	179.0 (3)
O2—C13—C14—C15	−179.9 (3)	C28—C29—C30—C31	0.9 (6)
C12—C13—C14—C15	1.2 (5)	C29—C30—C31—C26	0.0 (6)
C13—C14—C15—C10	−1.0 (5)	C27—C26—C31—C30	−0.4 (5)
C11—C10—C15—C14	0.6 (5)	C25—C26—C31—C30	177.6 (3)

### Hydrogen-bond geometry (Å, °)

**Table:**

*D*—H···*A*	*D*—H	H···*A*	*D*···*A*	*D*—H···*A*
N1—H1···N8	0.86 (4)	2.20 (2)	3.027 (5)	162 (4)
N5—H3···N4^i^	0.86 (3)	2.18 (3)	3.029 (5)	166 (3)

Symmetry codes: (i) *x*+1, *y*, *z*.
